# Environmental Burden Case Study of RFID Technology in Logistics Centre

**DOI:** 10.3390/s23031268

**Published:** 2023-01-22

**Authors:** Bibiana Bukova, Jiri Tengler, Eva Brumercikova, Frantisek Brumercik, Olga Kissova

**Affiliations:** 1 Faculty of Operation and Economics of Transport, University of Zilina, 010 26 Zilina, Slovakia; 2 Faculty of Mechanical Engineering, University of Zilina, 010 26 Zilina, Slovakia; 3 Institute of Lifelong Learning, University of Zilina, 010 26 Zilina, Slovakia

**Keywords:** RFID technology, environmental burden, e-waste

## Abstract

Radio Frequency Identification (RFID) technology has established itself as an effective tool for identifying various objects in all human and business areas. There are many studies describing the use of this technology. However, scientific articles only marginally address the issue of recycling or reusing radio frequency identifiers. Radio frequency identifiers are defined as electronic waste by European Union legislation. This article deals with the environmental burden resulting from the use of radio frequency identifiers in a selected logistics centre and courier company in the Slovak and Czech Republic territories. The research and its relevance have become topical in the context of pandemics and with the increasing demand for products and courier services. In order to access the level of the above-mentioned environmental burden in a relevant way, an analysis of the circulation of transport units (pallets) and radio frequency identifiers in the selected logistics centre was carried out. The research results showed that the selected logistics centre generated annually 5.7 t of the e-waste from radio frequency identifiers placed on received pallets. The amount of 139 kg of metal was present in the e-waste quantity. The partial results of the research were applied to the e-commerce area. This article’s conclusion is dedicated to the characterization of possibilities of reducing the environmental burden from the use of radio frequency identifiers in logistics.

## 1. Introduction

With the development of human society, the number of manufactured products has increased. With the increase in products, the requirements for their records increase too. The necessity of electronic records is caused by the wide use of information technologies that offer tools in the field of information processing [1,2]. Radio Frequency Identification (RFID) is a technology used for the identification of objects and information transfer from a distance through radio waves [3,4]. Radio frequency waves allow passage through many non-metallic materials.

RFID technology is currently one of the most widely used technologies for object identification and data transmission [5]. Application areas include material handling, supply chain management, manufacturing, product lifecycle management, and a wide range of different systems from healthcare to sports [6]. RFID technology is applied in a variety of fields such as agriculture [7,8], textile industry [9,10], healthcare [11], mechanical industry [12,13], or transport [14,15]. RFID technology is used in almost all industry branches, which is supported by the statistics of IDTechEx, exploring the RFID market for almost 20 years. Their RFID market research report named “RFID Forecasts, Players and Opportunities 2022–2032” provides a comprehensive overview covering passive (for UHF, HF, and LF frequencies), battery-assisted passive, active, and chipless RFID technologies, players, and markets. This report presents an unbiased analysis of primary data gathered through interviews with key players throughout the value chain (many of whom supply data to IDTechEx under nondisclosure agreements) as well as secondary data from all available sources, and it builds on expertise in the RFID industry. IDTechEx reported that the number of sold RFID tags showed a growing trend, starting with 17.5 billion in 2018, followed by 20 billion in 2019, 23.8 billion in 2020, and 28.4 billion in 2021 according to the available data from RFID forecasts. The largest number of those identifiers are the RFID tags in the UHF band. Experts foresee further significant growth in sales of RFID identifiers due to the development of Logistics 4.0 [16].

An RFID system, whatever its working frequency is, is composed of mandatory elements, such as RFID tags, RFID readers, and a software interface called “middleware”. Tags and readers are made up of elements, such as RFID chips and antennas [5]. RFID tags/labels are the most widely used type of RFID identifiers. Labels have imprinted, embossed, or otherwise fabricated radio frequency coils with a memory identifier. Their main disadvantage is their lower resistance to environmental influences [1].

In 2015, the authors conducted research investigating the effect of physical and electromagnetic factors on RFID identifiers that may occur during transport. The testing was conducted in the form of static and dynamic tests—both before and after the application of various physical factors, testing their durability and functionality. Simulations of external influences on RFID tags were carried out by changing the temperature acting on the tags, steaming the tags, or physically destroying the tags. Based on the results of the measured values, the greatest damage to the RFID identifier was detected under freezing conditions for a period of 72 h. Less negative damage was detected under freezing conditions for 24 and 48 h. Minor damage to the RFID identifier was observed when the RFID identifier was exposed to high temperatures from 40 to 60 °C for 1 h, with this value representing the normal temperature in containers and trailers during transport. Lower damage was also detected when the RFID identifiers were exposed to water, steam, and humidity [17].

It is necessary to monitor the environmental impact of RFID technology use. The authors focused on the environmental burden caused by the use of metals in RFID tags. The authors began investigating this problem in 2019 when they conducted research with RFID tags in households in the Slovak Republic. They focused primarily on the number of RFID tags, the types of RFID tags, and the types of products where RFID tags were placed. The authors found that RFID tags were thrown away by the monitored subjects in the municipal waste. After calculations, it was possible to conclude that Slovak households threw away about 7.867 tons of metal per year [18,19].

The main aim of this article is to warn and “highlight” the problem of RFID e-waste. In our opinion, this problem is hidden and will become much bigger in the future, because the issue of e-waste from passive RFID tags is not given enough attention. The increase of e-waste will continue to grow and, thus, can cause problems comparable to other types of small disposable products that society is already facing today.

## 2. Research Background

Based on the survey [18], the authors decided to investigate the environmental burden in a logistics centre (LC) where RFID technology is used most often for product identification. The largest logistics centre with its retail network in Slovakia was selected for the environmental burden model. The selected logistics centre uses one-time RFID stickers because of their operational properties and relatively low price. However, the waste is generated after their use too.

### 2.1. Logistics Centre Description

Logistics centres are high-capacity warehouses equipped with innovative technology, information, and communication systems. Their main activity is not only warehousing but, mainly, the consolidation of shipments. These processes include the distribution of larger deliveries, the mixing of goods according to the specific orders of individual business units, and the handling of goods (unloading, loading, and transfer within the warehouse). Based on this description, a logistics system of cross-docking is applied in the logistics centres [20,21].

The main benefit of logistics centres is the efficient supply of goods. In times of pandemics, their position was constantly increasing. For the suppliers of commercial establishments, this means facilitating the way of supply. The whole process is implemented in such a way that instead of the individual distribution of goods to each business unit separately, the goods are unloaded in one place only, i.e., in a logistics centre [22].

The logistics centre selected to research the environmental burden of RFID technology is one of the most modern and extensive in Slovakia. It was opened in 2017 in cooperation with a development company and a logistics operator. The logistics centre serves as the most important logistics hub for a logistics company in Central Europe. It has an area of almost 147,000 m^2^ and is part of one of the largest logistics parks in Slovakia. The described logistics centre operates three out of five large-scale warehouses located in the logistics park.

The logistics centre of the selected company focuses mainly on low-volume durable food, cosmetics, white and black goods, and clothing. The goods from this LC are distributed to sales units located in Slovakia, the Czech Republic, Hungary, and Poland. Private-label clothing is distributed not only to Central European countries but also to other trading partners. There are more than twenty-two such partners worldwide (e.g., in Germany, Saudi Arabia, and the United States). The benefit of this LC is to simplify supply chains, significantly reduce inventories in warehouses, and deliver goods to the business units in more precise quantities.

Since 2018, this LC has also acted as a consolidation centre for Central Europe. From this centre, the goods are distributed to logistics centres in Slovakia, Poland, Hungary, and Czech Republic. The consolidation centre is dedicated to the private labels of the selected company and food and nonfood goods from more than 100 suppliers. As a result, the number of new item groups in the LC distributed to all four countries has increased by more than 1000. In addition, the LC also serves as a tax warehouse for wines and liqueurs.

A detailed analysis of the LC is given in Table 1.

Table 1 shows data on the year-round daily movement (input and output) of pallet units in the LC. The annual input of pallets can be divided into two periods: January–June (1,448,800 pallets) and July–December (2,024,000 pallets). The annual output is 1,267,000 pallets in January–June and 1,932,000 pallets in July–December. These data form the basis for establishing the model of the annual environmental burden of RFID technology in this LC. The operation time of this centre is 365 days a year, and RFID technology is used to record the LC goods input and output.

### 2.2. Characteristics of the RFID Tag

The RFID tag sample was obtained from the LC and further investigated. This tag was marked as a measurement object with the following parameters:

- Frequency: UHF 860–960 MHz;
- International Standard: ISO/IEC 18000-63 Type C;
- Identifier packaging: paper, plastic film (antenna moulded in plastic film and then glued to the paper label);
- Antenna metal type: aluminium;
- Dimensions: 100 mm × 100 mm;
- Internal antenna dimension: 15 mm × 70 mm;
- Operating temperature: −40 °C to 85 °C (−40 °F to 185 °F) [23].

**Figure 1:**
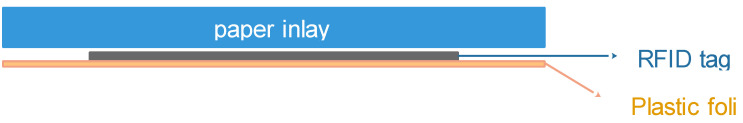
Scheme of the RFID identifier storage sample.

**Figure 2:**
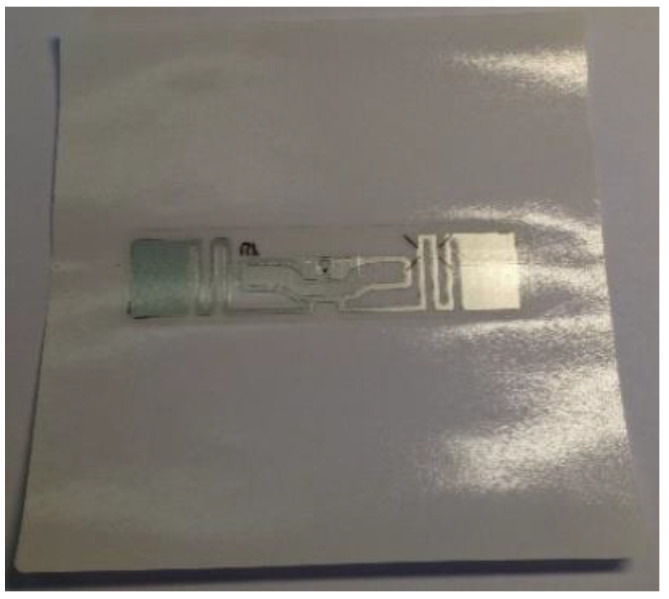
RFID identifier sample.

**Figure 3:**
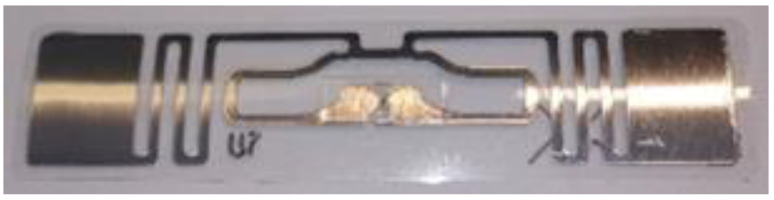
Partially extracted RFID identifier sample.

The scheme and the photo documentation of the RFID tag sample are shown in Figure 1, Figure 2 and Figure 3.

## 3. Research Methodology and Results

The mass characteristics of the investigated RFID identifier are shown in Table 2. It was necessary to extract the RFID tag to obtain the proportion of metal within the RFID identifier. The extraction process consisted of the separation of the RFID tag and the packaging made up of different materials (e.g., foil, paper, etc.). Special substances, tools, and software were used for the RFID tag extraction and measurement: e.g., digital weighing scale accurate to 0.001 g; C6000 solvent; surgical scalpel; SketchAndCalc area calculator software; scanner and precision rulers.

The data in Table 2 show that the average weight of the RFID tag is 1.654 g. The mean weight of the metal with foil is 0.161 g, which is approximately 9.73% of the total RFID tag weight. The mean weight of the pure metal was 0.040 g, which is approximately 2.42% of the total RFID tag weight (24.84% of the metal with foil weight). RFID tag manufacturers state on their websites that the proportion of metal in an RFID tag is no more than 20% [24].

### 3.1. Environmental Burden Model of RFID Technology in Logistics Centre

The starting point for the determination of the environmental load is the process of labelling the individual pallet units (PU). These pallet units at the LC input are equipped with RFID identifiers from the supplier. Since these RFID identifiers have no further use, they are destroyed and thrown away into the mixed waste at the LC (waste at the PU input). The output pallet units in the LC are tagged again before their placement into the individual sales distribution network with new RFID tags (waste at the PU output). The data of the identifier quantities at the input and output of the LC are presented in Table 3.

The data in the table show that 5.7 t of e-waste from RFID tags is generated annually in the selected LC by PU output. In this quantity, approximately 139 kg of metal is present. In the commercial operations of the LC by PU input, 5.3 t of RFID tags end up in waste annually, with a metal content of 128 kg. This is a model of the RFID tag load in the company’s commercial logistics operations considered only for pallet units. Based on this model of one LC retail chain, we concluded that 11 t of e-waste is generated annually in the logistics centre by its overall business operations with PU at the input and output. In this quantity, there is 267 kg of metal considering only the pallet units’ RFID tags. Due to the unavailability of the data, it was not possible to determine the share of the RFID tag waste in the LC’s total annual waste volume.

It is important to mention that further environmental burden is still generated by consumers because the products themselves are also tagged with RFID identifiers. This issue has already been discussed in an article on the environmental burden of RFID in households in the Slovak Republic [18].

It is also important to mention that the calculations were based on data from 2019. According to the available published data, the LC turnover in the Slovak Republic increased in 2021 on average by 20% [25].

### 3.2. Application of Environmental Burden Model of RFID Technology in E-Commerce

Due to the last pandemic and the increase in e-shopping, the sales volumes of e-stores increased significantly. This fact is associated with an increasing demand for logistics and shipping services, which ensure the delivery of goods to the consumer’s doorstep. The use of IT in logistics companies is a standard, but the increase in shipments also brings an increased need for fast and error-free processing of information, which makes the RFID technology possible, especially if used throughout the entire logistics chain.

In the presented example of the created environmental burden model of RFID technology application, the data published by a parcel delivery services company operating in Europe were used to determine the model of the annual environmental burden of RFID technology in the logistics chain (retailer–consumer). The company transported 44 million parcels in the Czech Republic in 2020. In 2021, it expected to increase its parcel traffic to 50 million items.

The data in Table 4 show that in 2020 and 2021, 94 million RFID tags can be expected in shipments to consumers only in the Czech Republic from e-shops cooperating with only this parcel delivery services company. The weight of the RFID “waste” was calculated according to the created model as 155.48 t, with a metal content of 3.76 t using the mean weight value of the RFID tags used in the examined logistics centre.

## 4. Discussion

The use of LCs and their services is not only increasing in traditional commerce, but logistics centres have an irreplaceable position in e-commerce. The increase in e-commerce performance is not only linked to the pandemic, but also to the globalisation of the industry and current consumer behaviour. For this reason, the model of the environmental burden of RFID tags was created and applied to an e-commerce business example. However, the companies described in this article are not the only producers of RFID e-waste. The main generation of RFID e-waste also includes traditional commercial establishments with their identification of partial products by various types of RFID tags.

With the current increase in the price of nonferrous raw materials, the question of recycling RFID tags is gaining even bigger importance. There are several possibilities for reducing the environmental burden of using RFID technology in commercial logistics:

- Use of industrial RFID tags in the logistics centres;
- Environmental labelling of RFID tags;
- Government financial support for innovative RFID recycling solutions.
- The first option aimed at the use of RFID identifiers in logistic centres is to place the RFID identifiers not on the packaging of the pallet unit, but on the pallet body. The placement of the tag should prevent damage to the label during fork handling. If plastic pallet units are used, it is advisable to use a pallet-embedded RFID identifier. This would ensure that the RFID identifier is reusable. However, this solution requires the use of a different type of RFID identifier (not a disposable one), which requires further investments. A pallet exchange system (pallet pooling) could also be used to overcome the growing environmental challenges.

**Figure 4:**
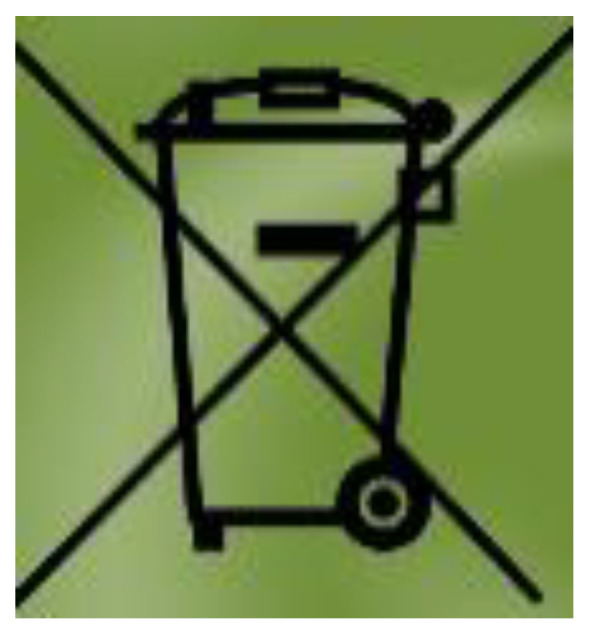
E-waste pictogram.

The second option for reducing the environmental burden focuses on visual reminders of e-waste for LC employees, business spaces, and also consumers. The solution is to properly segregate and handle the e-waste from the RFID tags. For better orientation for consumers, it would be advisable to label these tags by their manufacturers with a pictogram designed for e-waste (Figure 4).

Currently, there are conditions met in European cities and municipalities for the collection of household electrical waste. Residents have the following options for the disposal of household electrical waste:

- Point of sale collection;
- Collection point;
- Separate collection at least twice a year;
- Separate collection at a collection yard;
- Separate collection by mobile collection.

The last option to reduce the e-waste from RFID tags is to support the development and intensive use of technologies for their recycling and reuse, whereas at the beginning it would be advisable to focus at least on the RFID metal parts.

## 5. Conclusions

Consumers’ purchasing behaviour changes over time. This happens not only with the development of technology, and the use of artificial intelligence but also with the growth of environmental awareness. Demands on the quality of shopping are rising, logistics solutions are becoming more sophisticated, and the speed of processing the amount of information is optimizing global logistics chains. This trend cannot be stopped but only adjusted by “environmentalism”. The use of RFID technology in supply/logistics chains is justified. However, the recycling of RFID tags in global logistics needs to be addressed [19].

The production and operation of large numbers of RFID sensors will generate electronic waste and consume energy and materials. Until now, most of the population and industry do not address the problem of RFID as e-waste, because the tags are very small and the impact that they have on the environment is not perceived. Thus, they are not disposed of through the proper e-waste processing channel; instead, they are lost in the stream of generic waste [26]. If citizens and companies do not start separating the e-waste from RFID tags, the increase of e-waste will continue to grow and can cause problems comparable to other types of small disposable products that we are facing already today.

IDTechEx reports that 28.4 billion passive RFID tags were sold in 2021. The forecast of IDTechEx for the next few years states the growth of the RFID tags sale [16]:

- In 2023 to 31.841 billion passive RFID tags;
- In 2024 to 41.490 billion passive RFID tags;
- In 2029 to 102.330 billion passive RFID tags.

The increasing trend in the amount of e-waste is caused primarily by the following factors:

- Increasing goods consumption;
- Short life cycles of products;
- Lack of a circular economy in individual countries [27,28].

If the above-described factors cannot be suppressed, at the beginning of the waste reduction process, it would be sufficient to respect the applicable legislation. It is important to consistently implement Directive 94/62/EC of the European Parliament and of the Council of 20 December 1994 on packaging and packaging waste, where RFID tags are constituted as electronic waste (e-waste). Therefore, they are not allowed to be disposed of in municipal waste [29]. Unfortunately, this RFID disposal method is currently a widespread practice. In contrast with this fact, the applicable EU legislation sets out the waste management hierarchy with disposal in the last place as follows:

- Preventing the origin of e-waste;
- Preparation for re-use;
- Recycling;
- Another recovery, such as energy recovery;
- Disposal.

## Figures and Tables

**Table 1:** Characteristics of individual logistics centre warehouses.

Warehouse	LC 1	LC 2	LC 3
Area (m^2^)	26,509	64,639	55,840
Capacity (number of items)	6000	17,000	14,000
Number of loading bays	34	103	61
Total annual pallet input	3,472,000		
Total annual pallet output	3,199,000		

(Source: authors based on internal logistics centre documents).

**Table 2:** The results of weight measurement of the investigated RFID identifier.

Measurement Number	Weight (g)		
	Whole Tag	Metal with Foil	Metal
1	1.654	0.162	0.040
2	1.654	0.163	0.040
3	1.653	0.161	0.040
4	1.655	0.161	0.040
5	1.651	0.162	0.040
6	1.654	0.160	0.040
7	1.656	0.161	0.040
8	1.653	0.161	0.040
9	1.653	0.160	0.040
10	1.654	0.161	0.040
Mean value	1.654	0.161	0.040

(Source: authors).

**Table 3:** Data for determination of the annual weight of RFID tags used in the LC by PU operations.

Identifiers Classification	Quantity of Identifiers	Quantity of Identifiers	Annual Quantity	Weight of 1 Identifier	Weight of Metal in 1 Identifier	Total Weight of Identifiers	Total Weight of Metal in Identifiers
	January– June	July– December					
	(pcs)	(pcs)	(pc)	(g)	(g)	(g)	(g)
PU input	1,448,000	2,024,000	3,472,000	1.654	0.040	5,742,688	138,880
PU output	1,267,000	1,932,000	3,199,000	1.654	0.040	5,291,146	127,960
Total	2,715,000	3,956,000	6,671,000	1.654	0.040	11,033,834	266,840

(Source: authors).

**Table 4:** Assumption of the annual weight of RFID tags generated by parcel delivery services company operation.

Year	Annual Number of Unit Shipments	Weight of 1 Identifier	Weight of Metal in 1 Identifier	Total Weight of Identifiers	Total Weight of Metal in Identifiers
	(pc)	(g)	(g)	(g)	(g)
2020	44,000,000	1.654	0.040	72,776,000	1,760,000
2021	50,000,000	1.654	0.040	82,700,000	2,000,000
Total	94,000,000	-	-	155,476,000	3,760,000

(Source: authors).

## Data Availability

Not applicable.
